# Different subsets of tumor infiltrating lymphocytes correlate with NPC progression in different ways

**DOI:** 10.1186/1476-4598-9-4

**Published:** 2010-01-10

**Authors:** Yi-Lan Zhang, Jiang Li, Hao-Yuan Mo, Fang Qiu, Li-Min Zheng, Chao-Nan Qian, Yi-Xin Zeng

**Affiliations:** 1 State Key Laboratory of Oncology in Southern China, Sun Yat-sen University Cancer Center, 651 Dongfeng Road east, Guangzhou 510060, PR China; 2 Department of Experimental Research, Sun Yat-sen University Cancer Center, 651 Dongfeng Road east, Guangzhou 510060, PR China; 3 Department of Nasopharyngeal Carcinoma, Sun Yat-sen University Cancer Center, 651 Dongfeng Road east, Guangzhou 510060, PR China; 4 Department of Biotherapy, Sun Yat-sen University Cancer Center, 651 Dongfeng Road east, Guangzhou 510060, PR China

## Abstract

**Background:**

Increasing amounts of evidence indicate that tumor infiltrating lymphocytes (TIL) are correlated with the prognosis of cancer patients. This study focuses on the association between the densities of tumor infiltrating cytotoxic T lymphocytes (CTL), activated CTL, regulatory T lymphocytes (Treg) and Th17 lymphocytes, and the prognosis and clinicopathological features of nasopharyngeal carcinoma (NPC) patients.

**Results:**

Double immunohistochemical staining was performed in 106 biopsy specimens from newly diagnosed NPC patients. Prognostic values of infiltrating lymphocyte densities were evaluated by Kaplan-Meier analysis and Cox regression. The density of CD8+ TIL was positively correlated with lymph node metastasis, while the density of Foxp3^+ ^TIL was negatively associated with T stage (P < 0.05). For survival evaluation, the density of Foxp3^+ ^TIL or Foxp3^+ ^TIL combined with GrB^+ ^TIL together was associated with better overall survival (OS) and progression-free survival (PFS) (P < 0.01) in all patients and in the patients with late-stage diseases (Stages III and IV, P < 0.01). Meanwhile a low density of CD8^+^TIL or high ratio of FOXP3^+^TIL to CD8^+^TIL was correlated with better PFS in early stage patients (Stages I and II, P < 0.05). No significant association was found between IL-17^+ ^TIL and clinicopathological characteristic or survival of NPC patients.

**Conclusions:**

Our study identifies for the first time the tumor infiltrating Foxp3^+ ^TIL as an independent favorable factor in the prognosis of NPC patients, especially for the patients with late-stage diseases.

## Background

Nasopharyngeal carcinoma (NPC) is an epithelial neoplasm with high incidence in South China and South Asia, while with low incidence in most countries. There are three histological classficiation for NPC: Type 1, keratinising squamous-cell carcinoma; Type 2, Non- keratinising squamous-cell carcinoma; Type 3, Undifferetiation nasopharyngeal carcinoma. Due to the less symptoms and the difficulty to make clinical examination of nasopharynx, most patients with this disease are diagnosed only when the tumour at the advanced stage. Radiotherapy and concomitant chemoradiotherapy can improve overall survival and progression-free survival in NPC patients, but the prognosis remains poor in a significant number of patients with late-stage disease [1-4].

EBV infection positivity has been identified in most undifferentiated NPC (95%) by the expression of EBV latent phase antigens in the tumor cells including LMP1 (40-60%), LMP2, EBNA1 and BARF0. In addition, a large number of tumor infiltrating lymphocytes (TIL) are found around the NPC tumor tissues [5-8]. The majority of the cells in the tumor are reactive and consist predominantly of T cells with variable numbers of other inflammatory cells [9]. The presence of TIL correlates with an better prognosis in patients with several types of cancer, and each T lymphocyte subset has a unique role in the antitumor response [10-14]. Presence of tumor infiltrating cytotoxic T lymphocytes (CTL) has been linked to better patient survival in endometrial, ovarian, pancreatic and colorectal cancers [15-17]. Regulatory T cells (Treg) play an essential role in controlling immune responses to self and non-self antigens. It has been reported that the tumor infiltrating Treg cells are associated with a poorer prognosis in some cancers by suppressing the proliferation of effector T lymphocyte (i.e., cytotoxic T lymphocyte (CTL)) to prohibit an adequate tumor-specific immune response, thus enabling tumor growth [18-23]. In contrast, other researchers have reported that the tumor infiltrating Treg cells are either linked to a better prognosis or did not affect prognosis in some malignancies such as follicular lymphoma and squamous cell carcinoma [24-26]. However, the characteristics and functions of the TIL in NPC are still poorly understood. With advances in immunological techniques, adoptive immunotherapy is becoming a preferred choice with better tolerance. However, the clinical benefit of EBV-specific CTL-based adoptive immunotherapy is only observed in a small number of NPC patients, maybe due to tumor immune evasion or immune suppression of the tumor microenvironment [27,28]. Therefore it is important to understand the characteristics and functions of TIL and their relationship with tumor clinicopathological features and prognosis, as well as their potential role in immunotherapy of NPC.

In the present study, we aimed to evaluate the subsets of TIL including CTL, activated CTL, Treg, and Th17 cells by CD8/Foxp3, GrB/Foxp3 and Foxp3/IL-17 double IHC staining in 106 paraffin-embedded NPC tumor tissues. We correlated the densities of CD8^+ ^CTL, GrB^+ ^activated CTL, Foxp3^+ ^Treg and IL-17^+ ^Th17 cells with the clinical factors and outcomes in all NPC patients as well as in the patients with different disease stages. Furthermore, we evaluated the ratio of tumor infiltrating CD8+ cells to Foxp3^+ ^cells (the ratio of CD8/Foxp3) and Foxp3^+ ^cells together with GrB ^+ ^cells in predicting survival in all patients as well as in the patients with different disease stage.

## Results

### Patient Characteristics

Among the 106 patients, 84 (79.2%) were male, and the median age was 49 years (range: 22-73 years). Ninety-two patients (86.8%) were with histological type III (undifferentiated) NPC, 13 (12.3%) were with type II (non-keratinizing) NPC, and only 1 (0.9%) was with type I (squamous cell carcinoma) disease. Fifty-five (47.4%) of the tumor tissues were positively stained for EBV LMP1 antigen. Of these 106 patients, only 5 (4.7%) cases were with distant metastases before treatment, and 88 (83%) were with lymph node spreading diseases (N1-N3). Thirty eight (35.9%) patients were in early disease stages (I and II) while 68 (64.2%) were in late disease stages (III and IV) at initial diagnosis. From the total number of patients, 30 (28.3%) had dead and 37 (34.9%) presented with disease progression during the follow-up for five years. Detailed clinical information is shown in Table 1, and detailed clinical and treatment information for each patient is shown in additional file 1.

**Table 1:** Clinical characteristics of 106 patients with NPC

Characteristics	No. (%)
**Age, years**	
Median	49
Range	22-73
**Gender**	
Male	84 (79.2)
Female	22 (20.8)
**Tumor (T) stage**	
T1	24 (22.6)
T2	33 (31.1)
T3	33 (31.1)
T4	16 (15.1)
**Lymphoid Nodal (N) status**	
N0	18 (17.0)
N1	46 (43.4)
N2	36 (29.2)
N3	11 (10.4)
**Distant metastasis (M) status**	
M0	101 (95.3)
M1	5 (4.7)
**TNM stage**	
I	6 (5.7)
II	32 (30.2)
III	39 (36.8)
IVa+IVb	29 (27.4)
**Progression**	
No	69 (65.1)
Yes	37 (34.9)
**Death**	
No	76 (71.7)
Yes	30 (28.3)
Histological type*	
Type I	1 (0.9)
Type II	13 (12.3)
Type III	92 (86.8)
**LMP1**	
Positive	47 (44.3)
Negative	56 (52.8)
Missing	3(2.8)

* Type I: squamous cell carcinoma; Type II: non-keratinizing carcinoma; Type III: undifferentiated carcinoma

### The characteristics of the immunochemical variables and their relationship with NPC clinicopathological features

**Figure 1:**
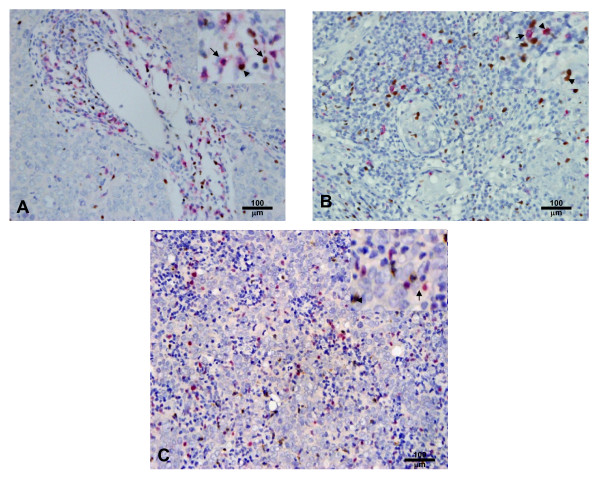
**Double immunohistochemical staining for CD8/Foxp3, Foxp3/GrB and Foxp3/IL-17**. A. NPC tumor tissue with numerous CD8^+ ^(red) and Foxp3^+ ^(brown) cells (× 200). B. NPC tumor tissue with numerous Foxp3^+ ^(brown) and GrB^+ ^(red) cells (× 200). C. NPC tumor tissue with numerous Foxp3^+ ^(red) and IL-17^+ ^(brown) cells (× 200). The staining patterns show that CD8 is on the cell membrane, GrB and IL-17 are in the cytoplasm, and Foxp3 is in the nucleus. There are a few CD8^+^Foxp3^+ ^and Foxp3^+^GrB^+ ^cells, but no Foxp3^+^IL-17^+ ^cells.

To characterize the subsets of TIL in NPC tissues, we defined the different lymphocyte subsets by specific antibodies: CD8 for CTL, Granzyme B (GrB) for activated CTL, Foxp3 for Tregs and IL-17 for Th17 cells. The CD8 immunostaining demonstrated cytomembrane staining in a subset of TIL around the tumor nests (Fig 1A). The median number of CD8+ cells was 52.50 ± 4.97 cells/high-power field (HPF) and the range was 0.80-231.30 cells/HPF. GrB immunostaining showed a cytoplasmic pattern (Fig 1B) in a subset of lymphocytes in tumor tissues. The median number of GrB^+ ^cells was 32.45 ± 3.01 cells/HPF, and the range was 0.40-160.60 cells/HPF. Foxp3 immunostaining demonstrated nuclear staining in a subset of lymphocytes around tumor tissues (Fig 1A, B, and 1C). The median number of Foxp3^+ ^cells was 87.23 ± 5.50 cells/HPF, and the range was 5.20-322.80 cells/HPF. IL-17 immunostaining established a cytoplasmic pattern (Fig 1C) in a small subset of lymphocytes around tumor tissues. The median number of IL-17^+ ^cells was 5.60 ± 2.36 cells/HPF and the range was 0.00-150.00 cells/HPF. The CD8/Foxp3 double positive subset (Fig 1A) showed a median number of 0.9 ± 0.16 cells/HPF and a range of 0.00-9.30 cells/HPF in TIL. The Foxp3/GrB double positive subset (Fig 1B) showed a median number of 1.8 ± 0.38 cells/HPF and a range of 0.01-22.80 cells/HPF in TIL (Table 2). By general logical linear regression analysis, no significant correlation was found between the densities of any two types of lymphocytes in the present study.

**Table 2:** Descriptive statistics of immunohistochemical variables

Variable*	Mean	SE	Median	Range
CD8^+ ^TIL	68.24	4.97	52.50	0.80-231.30
Foxp3^+ ^TIL	82.21	5.45	74.00	5.20-322.80
Granzyme B^+ ^TIL	39.98	3.01	32.45	0.40-160.60
IL17^+ ^TIL	14.75	2.36	5.60	0.00-150.00
CD8^+^Foxp3^+ ^TIL	1.40	0.16	0.90	0.00-9.30
Granzyme B^+ ^Foxp3^+ ^TIL	3.10	0.38	1.80	0.00-22.80

Note: *Number of TIL per field (×400).

To investigate the association between the density of different types of lymphocytes and clinical features in NPC patients, the median density of each type lymphocyte was used to separate the patients into high and low TIL groups. The density of CD8^+ ^TIL was positively associated with lymphoid nodal status in NPC patients (P = 0.038) while the densities of GrB and Foxp3-positive cells were negatively associated with the T stage (P < 0.01). The density of GrB^+^TIL was negatively associated with the age of patients (P = 0.011) and the density of Foxp3^+^TIL was negatively associated with NPC clinical stage (TNM stage, P = 0.043). No correlation was found between the density of IL-17^+ ^TIL and the NPC patient clinicopathological characteristics. No significant association was observed between any lymphocyte variables and LMP1 expression in NPC tumor cells (Table 3).

**Table 3:** Association of the CD8^+^, Granzyme B^+^, Foxp3^+ ^or IL17^+ ^TIL number and clinicopathological characteristics in NPC patients

Characteristics	CD8 Positive			Granzyme B Positive			Foxp3 Positive			IL17 Positive		
				
	≤52.50 (n = 53)	> 52.50 (n = 53)	*P*	≤32.45 (n = 53)	> 32.45 (n = 53)	*P*	≤74.00 (n = 53)	>74.00 (n = 53)	*P*	≤5.60 (n = 54)	>5.60 (n = 52)	*P*
**Age, years**												
≤49	27	27		21	34		24	31		27	28	
>49	26	25	0.846	32	19	0.011*	29	22	0.174	27	24	0.692
**Gender**												
Male	41	43		41	43		39	44		42	42	
Female	12	10	0.632	12	10	0.632	14	8	0.359	12	10	0.704
**Tumor (T) stage**												
T1+T2	28	29		22	35		21	36		26	31	
T3+T4	25	24	0.846	31	18	0.011*	32	17	0.003*	28	21	0.236
**Lymphoid Nodal (N) status**												
N0	13	5		9	9		10	8		9	9	
N1-3	40	48	0.038*	44	44	1.000	43	45	0.605	45	43	0.930
**TNM Clinical stage**												
I+II	21	17		16	22		14	24		15	23	
III+IVa+IVb	32	36	0.418	37	31	0.224	38	29	0.043*	39	29	0.077
**LMP1**												
-	25	31		30	26		28	28		31	25	
+	27	20	0.195	22	25	0.494	23	24	0.914	21	26	0.280

*P < 0.05, as determined by the Pearson chi^2 ^test.

No correlation was shown between the density of the subset of co-expression of GrB^+ ^and Foxp3^+ ^TIL and clinicopathological characteristics, however the density of CD8^+^Foxp3^+^TIL was positively associated with the lymphoid nodal status (P = 0.006) and increasing age (P = 0.041, Table 4). Finally, no co-expression of IL-17 and Foxp3 was found in NPC TIL by double immunohistochemical staining (data not shown).

**Table 4:** The association of the number of CD8^+ ^Foxp3^+ ^TIL, Foxp3^+ ^Granzyme B^+ ^TIL and the ratio of CD8^+ ^Foxp3^+ ^to CD8+ and GranzymeB^+ ^Foxp3^+ ^to Foxp3 TIL and clinicopathological characteristics in NPC patients

Characteristics	**CD8**^**+**^**Foxp3**^**+**^			**Granzyme B**^**+**^**Foxp3**^**+**^		
		
	≤0.90 (n = 53)	> 0.90 (n = 53)	*P*	≤1.80 (n = 54)	> 1.80 (n = 52)	*P*
**Age, years**						
≤49	24	31		26	29	
> 49	29	22	0.174	28	23	0.432
**Gender**						
Male	39	45		42	42	
Female	14	8	0.151	12	10	0.704
**Tumor (T) stage**						
T1+T2	25	32		29	28	
T3+T4	28	21	0.173	25	24	0.988
**Lymphoid Nodal (N) status**						
N0	14	4		10	8	
N1-3	39	49	0.010*	44	44	0.866
**TNM Clinical stage**						
I + II	18	20		19	19	
III+IVa+IVb	35	33	0.685	35	33	0.885
**LMP1**						
-	23	33		30	26	
+	27	20	0.098	22	25	0.494

Note. *P < 0.05, as determined by the Pearson chi^2 ^test.

### Immunohistochemical variables and survival evaluation

**Figure 2:**
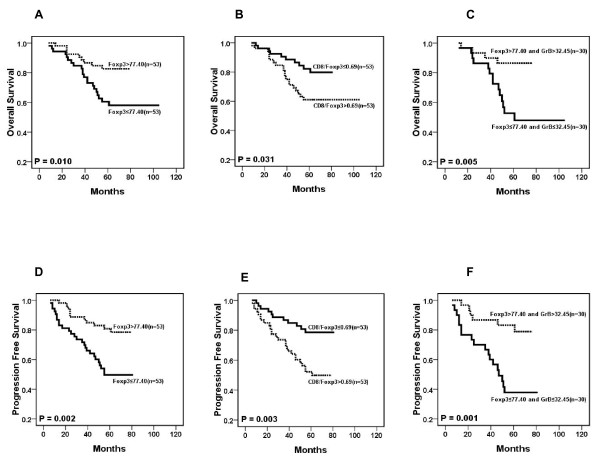
**Results of survival rate with immunohistochemical variables**. Individually, the number of Foxp3^+ ^cells (A and D), the ratio of CD8^+^TIL to Foxp3^+ ^TIL (B and E) and Foxp3 together with GrB^+ ^cells (C and F) are significantly associated with the overall survival (OS, P < 0.005) and progression-free survival (PFS, P < 0.005) in NPC patients.

Of these 106 NPC patients, Kaplan-Meier and log-rank test analyses indicated that the groups with Foxp3^+^TIL>77.2 cells/HPF (median, n = 53) had a significantly better overall survival (OS, 65 vs. 56 months, median/5 year, P = 0.01) and progression-free survival (PFS, 64 vs. 52 months, median/5 year, P = 0.002) when compared to the groups with Foxp3^+^TIL = 77.2 cells/HPF (median, n = 53) (Fig 2). However, the number of CD8^+^, GrB^+ ^or IL-17^+ ^TIL is not significantly associated with OS or PFS (data not shown). Upon univariate analysis, age, sex, LMP1 expression, and densities of CD8^+^TIL, GrB^+^TIL and IL-17^+^TIL showed no prognostic significance for OS and PFS. In contrast, tumor size, lymphoid nodal status, TNM clinical stage and the density of Foxp3^+^TIL were predictors for OS and PFS (Additional File 2). Furthermore, the multivariate Cox proportional hazards analysis of covariates displayed *P *< 0.05 in univariate analysis (tumor size, lymphoid nodal status, TNM clinical stage and the number of Foxp3^+^TIL) indicating that after backward elimination, Foxp3 becomes a strong independent favorable prognostic factor for OS and PFS (P < 0.01, Table 5).

**Table 5:** Multivariate analysis of factors and Foxp3^+ ^TIL associated with survival

Variables	OS (n = 106)			PFS (n = 106)		
		
	Hazard Ratio	95% CI	*P*	Hazard Ratio	95% CI	*P*
Tumor (T) stage (4+3/2+1)	0.803	0.331-1.949	0.627	0.930	0.408-2.119	0.863
Nodal (N) status (3+2+1/0)	5.181	0.679-40.071	0.115	3.880	0.886-16.994	0.072
TNM stage (III+IVa+IVb/I+II)	2.582	0.798-8.351	0.113	1.704	0.622-4.674	0.300
Foxp3^+ ^TILs (high/low)	0.378	0.170-0.841	0.017*	0.331	0.161-0.682	0.003*

NOTE: The Cox proportional hazards regression model was used in univariate analysis.

The multivariate Cox proportional hazards assessment indicated that the ratio of CD8 to Foxp3 is an independent feature in the PFS survival of patients (RH = 2.39, P = 0.016) and the combination of Foxp3 and GrB is also an independent prognostic factor in NPC (Table 6). The high expression of Foxp3 and GrB correlated with better OS (66 vs. 51 months, median/5 year) and PFS (64 vs. 46 months, median/5 year) compared to low expression of Foxp3 and GrB (RH<1, P < 0.01) (Fig 2).

**Table 6:** Multivariate analysis of factors for the ratio of CD8^+ ^TIL to Foxp3^+ ^TIL and Foxp3^+ ^TIL combined with Granzyme B^+ ^TIL with survival

Variables		OS (n = 106)			PFS (n = 106)		
		
		Hazard Ratio	95% CI	*P*	Hazard Ratio	95% CI	*P*
**The ratio of CD8 to Foxp3**							
Tumor (T) stage (4+3/2+1)		1.007	0.419-2.420	0.988	1.185	0.523-2.686	0.685
Nodal (N) status (3+2+1/0)		4.172	0.545-31.939	0.169	2.821	0.655-12.154	0.164
TNM stage (III+IVa+IVb/I+II)		2.444	0.754-7.928	0.136	1.561	0.569-4.286	0.387
CD8/Foxp3 (high/low)		1.898	0.885-4.071	0.100	2.393	1.174-4.874	0.016*
**Combination of Foxp3 and GrB**							
Tumor (T) stage (4+3/2+1)		0.647	0.256-1.639	0.359	0.750	0.318-1.768	0.510
Nodal (N) status (3+2+1/0)		5.798	0.757-44.421	0.091	4. 681	1.080-20.280	0.039*
TNM stage (III+IVa+IVb/I+II)		3.078	0.940-10.075	0.063	2.093	0.759-5.78	0.153
**Combination of Foxp3^+ ^and Granzyme B^+ ^TIL**							
Overall		NA		0.028*	NA		0.001*
Foxp3GrB high VS Foxp3GrB low		0.314	0.111-0.887	0.029*	0.197	0.072-0.540	0.002*

Note: *significant. Abbreviations: CI, confidence interval; OS, overall survival; PFS, progression-free survival; NA, not applicable.

### Immunohistochemical variables and survival evaluation in the patients with different disease stages

**Figure 3:**
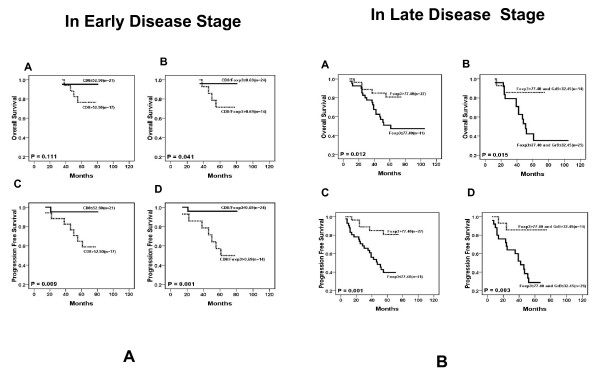
**Results of survival rate with immunohistochemical variables in different disease stages (n = 39)**. A. In the early disease stage, the number of CD8^+ ^cells (A and C) and the ratio of CD8^+^TIL to Foxp3^+ ^TIL (B and D) are significantly associated with PFS (P < 0.05). B. In the late disease stage, the number of Foxp3^+ ^cells (A and C) and Foxp3^+ ^together with GrB^+ ^cells (B and D) are significantly associated with OS (P < 0.01) and PFS (P < 0.01).

To further evaluate the association between different lymphocyte variables and the patient outcome, we classified the patients by their clinical stages: early disease (stages I and II, n = 38) and late disease (stages III and IV, n = 68). We assessed the correlation between the density of CD8, GrB, Foxp3 and IL-17 positive TIL and patient outcome in different disease stages. Our results showed that, in early disease stage patients, a higher number of CD8^+^TIL was significantly associated with poor PFS (P = 0.025) without significant association with OS. A higher ratio of CD8 to Foxp3 was also found to be significantly associated with poor OS and PFS (P = 0.041 and 0.001, respectively, Fig 3A). However, the aforementioned factors did not significantly impact OS or PFS in late stage patients (data not shown). In contrast, a higher Foxp3^+^TIL number was significantly associated with a better OS (P = 0.012) and PFS (P = 0.001) in late stage patients (Fig 3B), but had no significant influence on OS and PFS in early stage patients. Additionally, the combination of tumor infiltrating Foxp3^+ ^cells and GrB^+ ^activated CTL was associated with the survival of late stage patients: a higher number of Foxp3^+ ^and GrB^+ ^cells was associated with a longer OS and PFS compared to a lower number of Foxp3^+ ^and GrB^+ ^cells (P < 0.001, Fig 3B).

## Discussion

To better understand the special immunological state of the inflammatory background in NPC and its contribution to the clinicopathological characteristics and prognosis of NPC patients, we characterized the T cell infiltrating lymphocytes in a series of cases by immunohistochemical staining with antibodies specific to CTL, activated CTL, Treg and Th17 subsets. We have demonstrated that the number of Foxp3^+^TIL (Treg) was significantly associated with a favorable outcome (OS and PFS) in Chinese NPC patients (Fig 2); this association was correlated with the disease stage since Foxp3 was a favorable factor for survival in late stage patients but no significant influence on the early stage patients (Fig 3B, P < 0.01). Furthermore, multivariate Cox proportional hazards analysis indicated that Foxp3 was a strong favorable prognostic factor for OS and PFS for all patients (HR = 0.233, P < 0.01, Table 5). Our result is in line with published reports that the Foxp3^+^TIL is positively associated with better survival in EBV-associated classic Hodgkin lymphoma and follicular lymphoma [29,30]. Due to their ability to suppress naïve T cells and effector T cells, Treg cells are usually defined as immune suppression cells that inhibit antitumor immunity and help tumor cell immune evasion. Some studies even found that the number of tumor infiltrating Foxp3^+^Treg cells was completely associated with poor survival in several kinds of solid carcinoma such as ovarian and cervical cancers [15,31-34]. Since it is difficult to define the general function of Treg cells in all types of cancer, we suggest that the function of Treg in the tumor microenvironment should be specifically analyzed in different malignancies. Undifferentiated NPC is associated with EBV infection. The virus antigens expressed in the tumor cell are neo-antigens recognized by host immune cells, so many different lymphocytes are homing to tumor tissues including CD8+ T cells, CD4+ T cells and other immune cells. The possible role of Tregs in controlling immune response in the NPC microenvironment to prevent lymph node spreading of the cancer cell is worthy of recognition. A recent study also demonstrated that the Foxp3^+ ^Treg cells could facilitate early immune responses to local viral infection at least in part by orchestrating a timely homing of immune effector cells to the site of infection [35]. In our study, the ratio of CD8^+^TIL to Foxp3^+^TIL (CTL/Treg cells) was significantly associated with poor OS and PFS in early stage patients (Fig 3A), but had no effect on late stage patients, implying that increased number of Foxp3^+^Treg cell together with decrease number of CD8^+^CTL could suppress tumor growth and lymph node metastasis at the early-stage of NPC (high CD8^+^TIL number is positively associated with N stage in Table 3). There are three types of Treg including thymic-derived naturally-occurring Tregs (nTreg), antigen-induced Tregs (iTreg) and TGFβ+ Treg cells (Th3). The mechanisms of suppressing proliferation of naïve or effector T cells of these Treg cells are controlled by cell-to-cell contact or secreting cytokines IL-10 and TGFβ [36-42]. However, it has been reported that these Treg cell subpopulations are unstable in vivo and able to shift to IL-17 secreting cells[4,43,44]. Moreover, the anti-tumor role of these subset cells has not been clarified. However, it has been identified that some tumor derived Treg cells with the tumor antigen specificity could recognize the autologous antigen specific tumor cell and secret IFNγ *in vitro *[45]. In the present study, we found that a high density of Treg (Foxp3^+^TIL) was a favorable factor, while the increased number of CD8^+ ^CTL was not a favorable factor in NPC patients. We are currently evaluating the functions and related mechanism of Foxp3^+^Treg cell derived from NPC TILs by *in vitro *and animal experiment.

The cytotoxic T cell (CTL) has been assigned an important role in antitumor immunity, but in our results the CD8^+ ^CTL density was positively associated with poor PFS (Fig 3A) in early stage patients, and with the lymphoid nodal spreading (Table 3). This result was unexpected; however, we have previously found that the CD8^+^TIL from NPC patients failed to release IFNγ by the stimulation of autologous EBV-specific antigen cells in vitro [46]. Therefore, a possible explanation for this result is that a CD8^+ ^CTL function is impaired thus the CD8^+ ^CTL could not kill the tumor cell in NPC TIL. Another possibility is that the acute immune response could induce the spreading of tumor cells to the regional lymphoid node. To further evaluate the function of tumor infiltrating CTL in NPC, we detected Granzyme B positive activated CTL in TIL. In our data, the The number of activated CTL (GrB^+^TIL) is negatively associated with tumor stage (the same as Treg) and significantly decreased in the senior age patient group (P < 0.01, Table 3). However, the higher number of activated CTL seems to correlate with better OS and PFS in total NPC patients (P = 0.14 and 0.10, respectively) and in late disease stage patients (P = 0.19 and 0.08, respectively) although no statistical significance was found (data not shown). Our further evaluation of the correlation and imbalance of CTL and Treg cell in NPC TIL showed that the ratio of CD8/Foxp3 was significantly associated with poorer OS and PFS in early stage patients (P = 0.04 and 0.001, respectively). When we evaluated the impact of the combination of Treg and activated CTL densities, our results showed that the higher density of Treg and activated CTL was associated with better survival in total and late stage patients (OS P = 0.005 and 0.015, respectively; PFS P = 0.001 and 0.003, respectively). One published report in 2002 showed that the number of activated CTL was associated with a rapid fatal outcome in 43 NPC patients [47]; another published report in 2009 showed that the number of Treg cells was increased while the expression of the ζ-chain decresed in NPC tumor tissues compared to normal nasopharynx tissues[48]. Our results seem to contradict these finding, but the discrapensy may be due to differences in sample sizes and racial origins.

In order to understand the role of the CD8^+^Foxp3^+^Treg and GrB^+^Foxp3^+ ^activated Treg cell subsets in NPC tumor tissues, we double stained CD8 and Foxp3, and GrB and Foxp3. The CD8^+^Foxp3^+ ^and GrB^+^Foxp3^+ ^TIL were in small number (1.39 ± 0.9 cells/HPF and 3.52 ± 2 cells/HPF, respectively) compared to the total TIL. We found that the number of CD8 and Foxp3 double positive TIL was positively correlated with tumor lymphoid nodal metastasis in NPC patients (P = 0.006) (the same as CD8^+^TILs). However, no correlation between GrB^+^Foxp3^+^TIL and clinicopathological characteristic NPC patients was found (Table 4). Although many publications have showed that the expression of Granzyme B is a marker of activated Treg and the activity of Treg is dependent on the Granzyme B pathway [49,50], it remains difficult to define the function of the GrB^+^Foxp3^+ ^subset in NPC.

In total human CD4^+ ^T cells, in addition to the traditional Th1 and Th2 helper T cell subsets, Treg and Th17 cell populations have also been identified. The Th17 cell is newly characterized by secretion of IL-17, IL-22, and IL-21 with or without IFN-γ, and its function is opposite to that of the Treg cell in autoimmune disease since Th17 cells usually promote inflammatory processes [51-54]. The characteristic of Th17 cells in NPC is unknown. We therefore detected the IL-17^+^TIL by double staining of IL-17 and Foxp3. We could not find IL-17^+^Foxp3^+^TIL by IHC staining in vivo, although there are several recent reports of the in vitro detection of Il-17 and Foxp3 double positive cells in human [4,55]. We also found IL-17-secreting T clones highly expressing Foxp3, and with the suppression function to naïve T cells from NPC TIL in vitro (unpublished data), the failure to find the double IL-17 and Foxp3 positive TIL in NPC may be due to the lack of sensitivity of the IHC staining method or the differences between in vivo and in vitro conditions. In our study, no correlation between the number of IL-17^+^TIL and the clinicopathological characteristics was found (Table 3). We also could not find a significant association between the number of IL-17^+^TIL and outcome of patients by univariate analysis (See additional file 2) although it seems that a higher number of IL-17^+^TIL was associated with better OS and PFS of patients (P = 0.19 and 0.39, data not shown); however, this association was not statistically significant. Recently, Zou and colleagues also reported that in ovarian cancer the number of Th17 cells was associated with a better outcome [56].

## Conclusions

Our present study found that the tumor infiltrating Foxp3^+ ^Treg cell was an independent favorable factor for the T stage and for survival in the NPC patients, while the tumor infiltrating CD8^+^, GrB^+ ^or IL-17^+ ^cells was not an independent factor for survival. Furthermore, the ratio of CD8^+^TIL to Foxp3^+^TIL was significantly associated with poor OS and PFS in all NPC patients and poor PFS in the patients with early stage diseases, and was an independent factor for PFS in multivariate analyses. Our results support the assumption that the Treg cell but not the CTL take a favorable role in anti-tumor immunity of the NPC patients. These could be explained by the impaired function of CTLs in NPC patients. Our finding that the combination of Foxp3^+^TIL and GrB^+^TIL (activated CTL) was an independent favorable factor and was significantly associated with improved outcomes of OS and PFS in the patients with late-stage disease further support the assumption of the favorable role of Treg in NPC patients. In conclusion, our study discloses contrasting roles of tumor infiltrating lymphocyte subsets in the immunomodulation of NPC, and gives some support for the new strategy choice of immunotherapy for NPC.

## Methods

### Tissue specimens

Formalin-fixed, paraffin-embedded tissues from 106 NPC patients were used. The NPC biopsy specimens were collected between 2002 and 2003 at the Sun Yat-sen University Cancer Center. Adequate clinical follow-up data were available in these patients. NPC were classified histologically into three types according to WHO classification: squamous cell carcinoma (SCC, type I), non-keratinizing carcinoma (NKC, type II) and undifferentiated carcinoma (UC, type III). The disease stage of each patient was classified by the 2002 AJCC staging system [57]. The characteristics of the NPC patients are shown in Table 1. This study was conducted in accordance with the Helsinki Declaration, and all patients signed a consent form approved by the Research Ethics Committee of the Sun Yat-sen University Cancer Center.

### Immunohistochemistry

The paraffin-embedded tissues were sectioned continuously at a thickness of 4 μm. The co-expression of FoxP3 with CD8 and of Granzyme B with IL-17 were detected by sequential double-immunohistochemical staining according to the instructions of the double-staining EnVison™ G/2 Doublestain System (DakoCytomation, Glostrup, Denmark), or using the EnVison™ Detection Kit (DakoCytomation, Glostrup, Denmark) for LMP1. In brief, the tissue sections were deparaffinized and then rehydrated through graded alcohols, sections were immersed into preheated EDTA (PH 8.0). Antigen retrieval were performed by microwave (95°C for 10 min for LMP1; 95°C for 18 min for other antigens), and then allowed to cool to room temperature. Endogenous peroxidase and alkaline phosphatase activities were blocked with dual endogenous enzyme block reagent from the doublestain kit, and nonspecific binding sites were blocked with goat serum at room temperature for 20 min. Primary antibodies including mouse monoclonal anti-EBV LMP1(Clone CS.1-4, DAKO, Glostrup, Denmark; diluted at 1:80), mouse anti-human CD8(Clone CD8/144B, DAKO, Glostrup, Denmark; diluted at 1:40), mouse anti-human Granzyme B (clone GrB-7, Glostrup, Denmark; diluted at 1:25), mouse anti-human Foxp3(Clone 221D/D3, Santa Cruz Biotechnology, CA, USA, diluted at 1:100), and goat monoclonal anti-human IL-17(R&D Systems, Abingdon, UK; diluted at 1:300) were incubated at 4°C overnight, and developed with peroxidase and 3,3'-diaminobenzidine tetrahydrochloride (brown), or permanment red (red).

Data were obtained by counting the positively stained lymphocytes in 5 separate 400× high-power microscopic fields (HPFs) and calculating the mean number of positively stained cells per HPF. Mouse IgG1 (DAKO) and normal goat IgG (Santa) negative control stains were generated and evaluated.

### Statistical Analysis

All data analysis was carried out with the SPSS 13.0 software (SPSS, Chicago, IL, USA). The median value was used to cut off the subgroups of all immunohistochemical variables in our data. The Pearson chi^2 ^test was carried out to assess the relationship among lymphocytic variables and clinicopathological characteristics. Two-tailed P < 0.05 was judged to be significant.

Overall survival (OS) was defined as the period from initial diagnosis to death by any cause; progression-free survival was defined as time from initial diagnosis to the progression of NPC or death by any cause. Cumulative survival time was calculated by the Kaplan-Meier method and analyzed by the log-rank test. Univariate and multivariate analyses were based on the Cox proportional hazards regression model.

## Abbreviations

The abbreviations used are: NPC: Nasopharyngeal Carcinoma; TIL: Tumor infiltrating lymphocytes; GrB: Granzyme B; Foxp3: Forkhead Box P3; CTL: Cytotoxic T lymphocytes; Treg: Regulatory T cells; OS: Overall survival; PFS: Progression-free survival; IHC: Immunohistochemical staining; HPF: High-power field; SSC: squamous cell carcinoma; NKC: non-keratinizing carcinoma; UC: undifferentiated carcinoma; VS: versus; CI: confidence interval; NA: not applicable.

## Competing interests

The authors declare that they have no competing interests.

## Authors' contributions

LNZ performed the immunohistochemical staining and stastical analysis; JL designed the experiment, analyzed the data and wrote the manuscript; HYM and FQ collected the samples and clinic data; LMZ and CNQ modified and revised the manuscript; YXZ supervised in the design of the study and finalized the manuscript. All authors read and approved the final manuscript.

## Supplementary Material

Additional file 1 **The record of experiment and clinical data for each NPC patient**. All results for Description: immunochemical variables and clinical information, fellow-up data for each patient were shown in this excel file. Click here for file

Additional file 2 **Univariate analyses of factors associated with survival of patients**. The Description: univarate statistic analyses for the association of age, gender, TNM stage and immunochemical variables including LMP1, CD8, GrB, Foxp3 and IL-17 and the survival of patients. Click here for file
