# Supplementary material for: Different subsets of tumor infiltrating lymphocytes correlate with NPC progression in different ways
Source: Mol Cancer. 2010 Jan 10;9:4. doi: 10.1186/1476-4598-9-4 (PMC2818695; doi:10.1186/1476-4598-9-4)
Supplement: Additional file 2 — Univariate analyses of factors associated with survival of patients. The Description: univarate statistic analyses for the association of age, gender, TNM stage and immunochemical variables including LMP1, CD8, GrB, Foxp3 and IL-17 and the survival of patients. [file 1476-4598-9-4-S2.DOC]

**Univariate analyses of factors associated with survival**

| Variables | OS(n=116) | | |  | PFS(n=116) | | |
| --- | --- | --- | --- | --- | --- | --- | --- |
|  | Hazard Ratio | 95% CI | *P* |  | Hazard Ratio | 95% CI | *P* |
| Age, years (≤48/>48) | 1.935 | 0.920-4.070 | 0.082 |  | 1.222 | 0.640-2.334 | 0.544 |
| Gender (Male/Female) | 0.736 | 0.282-1.923 | 0.532 |  | 0.882 | 0.387-2.009 | 0.765 |
| Tumor (T) stage (1/2/3/4) | 1.445 | 1.003-2.081 | **0.048*** |  | 1.408 | 1.009-1.964 | **0.044*** |
| Nodal (N) status (0/1/2/3) | 2.255 | 1.491-3.412 | **0.000*** |  | 1.898 | 1.310-2.749 | **0.001*** |
| TNM stage (Ⅰ+Ⅱ/Ⅲ+Ⅳa+Ⅳb) | 3.527 | 1.348-9.226 | **0.010*** |  | 2.500 | 1.141-5.479 | **0.022*** |
| LMP1 (negative/positive) | 0.852 | 0.403-1.801 | 0.675 |  | 0.739 | 0.376-1.454 | 0.381 |
| CD8+ TIL (low/high) | 1.251 | 0.607-2.575 | 0.544 |  | 1.611 | 0.829-3.130 | 0.160 |
| Granzyme B+ TILs (low/high) | 0.577 | 0.274-1.213 | 0.147 |  | 0.574 | 0.295-1.117 | 0.102 |
| Foxp3+ TILs (low/high) | 0.243 | 0.099-0.595 | **0.002*** |  | 0.251 | 0.114-0.549 | **0.001*** |
| IL17+ TILs (low/high) | 0.617 | 0.297-1.282 | 0.195 |  | 0.753 | 0.393-1.444 | 0.393 |

Note. * means significant
